# No causal effect of genetically determined circulating homocysteine levels on psoriasis in the European population: evidence from a Mendelian randomization study

**DOI:** 10.3389/fimmu.2023.1288632

**Published:** 2023-11-08

**Authors:** Chaojian Chen, Shuo Liu, Junhao Liu, Ziqi Zheng, Yixi Zheng, Zhongliang Lin, Yuchun Liu

**Affiliations:** ^1^ Department of Clinical Medicine, Shantou University Medical College, Shantou, China; ^2^ The Intensive Care Unit, Jieyang People’s Hospital, Jieyang, China

**Keywords:** homocysteine, psoriasis, Mendelian randomization, casual effect, genome-wide association studies

## Abstract

**Background:**

Although numerous studies demonstrated a link between plasma homocysteine (Hcy) levels and psoriasis, there still exists a certain level of controversy. Therefore, we conducted a Mendelian randomization study to investigate whether homocysteine plays a causative role in the development or exacerbation of psoriasis.

**Methods:**

A two-sample Mendelian randomization (MR) analysis was conducted. Summary-level data for psoriasis were acquired from the latest R9 release results from the FinnGen consortium (9,267 cases and 364,071 controls). Single nucleotide polymorphisms (SNPs) robustly linked with plasma Hcy levels at the genome-wide significance threshold (*p* < 5 × 10^−8^) (18 SNPs) were recognized from the genome-wide meta-analysis on total Hcy concentrations (*n* = 44,147 participants) in individuals of European ancestry. MR analyses were performed utilizing the random-effect inverse variance-weighted (IVW), weighted median, and MR-Egger regression methods to estimate the associations between the ultimately filtrated SNPs and psoriasis. Sensitivity analyses were conducted to evaluate heterogeneity and pleiotropy.

**Results:**

MR analyses revealed no causal effects of plasma Hcy levels on psoriasis [IVW: odds ratio (OR) = 0.995 (0.863–1.146), *p* = 0.941; weighed median method: OR = 0.985 (0.834–1.164), *p* = 0.862; MR-Egger regression method: OR = 0.959 (0.704–1.305), *p* = 0.795]. The sensitivity analyses displayed no evidence of heterogeneity and directional pleiotropy, and the causal estimates of Hcy levels were not influenced by any individual SNP.

**Conclusion:**

Our study findings did not demonstrate a causal effect of genetically determined circulating Hcy levels on psoriasis.

## 1 Introduction

Psoriasis is a common inflammatory skin disease manifested as distinct red patches covered in silvery scales (1). Globally, over 60 million people suffer from psoriasis with varying prevalence across different regions. It has been reported that Oceania had the highest prevalence, followed by Western Europe, Central Europe, North America, and East Asia (2). Research also found that white individuals exhibited higher morbidity rates compared with other ethnic populations (3, 4). Children were reported to have lower prevalence and incidence rates than adults (5, 6). The differences in psoriasis prevalence and morbidity can be attributed primarily to the genetic makeup of individuals (7). Through the latest genome-wide association studies (GWASs), researchers have recognized more than 80 psoriasis susceptibility loci, which accounted for approximately 30% of the overall heritability (8).

Actually, psoriasis is not only a skin disease but is also associated with the occurrence of many other diseases including rheumatological, cardiovascular, and inflammatory bowel diseases and metabolic syndrome (9). In recent years, the relationship between psoriasis and its associated disorders has gradually become the research focus, especially for cardiovascular diseases like ischemic stroke, heart failure, myocardial infarction, and atrial fibrillation (10–13). Increasing attention has been paid to homocysteine (Hcy), with its elevated levels in the blood being identified as an independent risk factor for the occurrence of cardiovascular diseases (14, 15). Accordingly, the association between Hcy and psoriasis has drawn considerable attention.

Previous studies have found that patients with psoriasis tended to exhibit significantly higher plasma Hcy levels compared with controls (16–26), some of which also uncovered the association between the severity of psoriasis and circulating Hcy levels, possibly by affecting inflammation and oxidative stress pathways (17, 22, 23). However, some studies demonstrated that no significant differences in Hcy levels existed between patients with psoriasis and healthy individuals (27–30). It is important to note that research in this field is not entirely conclusive and the exact nature of the relationship between Hcy and psoriasis is not fully understood. It is of great interest and significance to investigate whether there is a link between hyperhomocysteinemia and psoriasis. More studies are required and reliable methods need to be employed in research to establish a definitive link and determine the role of Hcy in the development of psoriasis.

Mendelian randomization (MR), a novel epidemiological research method, can be adopted to investigate the causal relationship between exposure and outcome. It has been demonstrated as a dependable approach employing genetic variants to effectively and comparably group individuals based on their genotypes, akin to a randomized trial’s random allocation process (31, 32). Consequently, the challenges posed by confounding and reverse causation, commonly encountered in observational studies, can be circumvented, which enables the estimation of the causal impact of a risk factor on a particular outcome of interest (31–33). Previous research has explored the causal association between psoriasis and alcohol consumption (34), smoking (34), body mass index, vitamin D levels (35, 36), serum lipids (37, 38), COVID-19 (39), inflammatory bowel diseases (40), cardiovascular diseases (10, 41), non-alcoholic liver diseases (42), ankylosing spondylitis (43), and lung cancer (44, 45) via the MR approach. However, there is a scarcity of research investigating the causal effect of plasma Hcy levels on psoriasis. Therefore, we conduct this study adopting MR methods to investigate whether homocysteine contributes causally to the development or exacerbation of psoriasis.

## 2 Materials and methods

### 2.1 Study design

**Figure 1:**
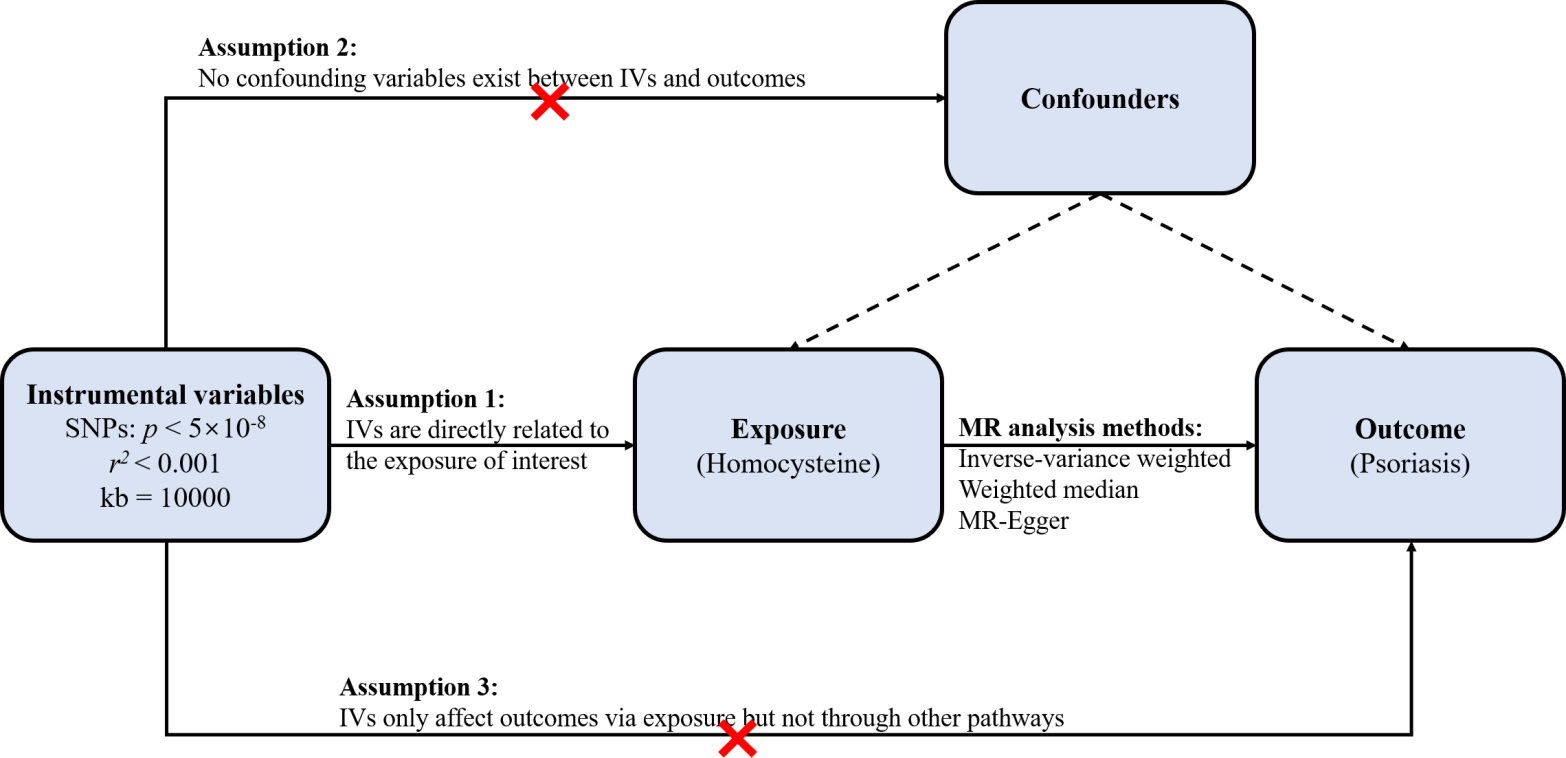
Schematic representation of the two-sample Mendelian randomization (TSMR) analysis.

We conducted a two-sample Mendelian randomization (TSMR) analysis to evaluate the causal relationship between circulating Hcy levels and psoriasis. In MR analysis, multiple single nucleotide polymorphisms (SNPs) representing genetic variation chosen as instrumental variables (IVs) should satisfy three crucial assumptions (**Figure 1**): Firstly, IVs are directly related to the exposure of interest; secondly, there exist no confounding variables between IVs and outcomes; and thirdly, IVs only influence outcomes via exposure but not through other pathways (46, 47).

### 2.2 Outcome data sources

There are publicly accessible databases resulting from genome-wide association studies, one of which is the FinnGen research project that offers genetic insights from a well-phenotyped isolated population (48). Summary-level data for psoriasis were acquired from the most recent R9 release results from the FinnGen consortium (https://r9.finngen.fi/). The database comprised 9,267 cases with a diagnosis of psoriasis based on the ICD-10 (International Classification of Diseases) diagnostic criteria and 364,071 controls.

### 2.3 Selection of IVs

**Figure 2:**
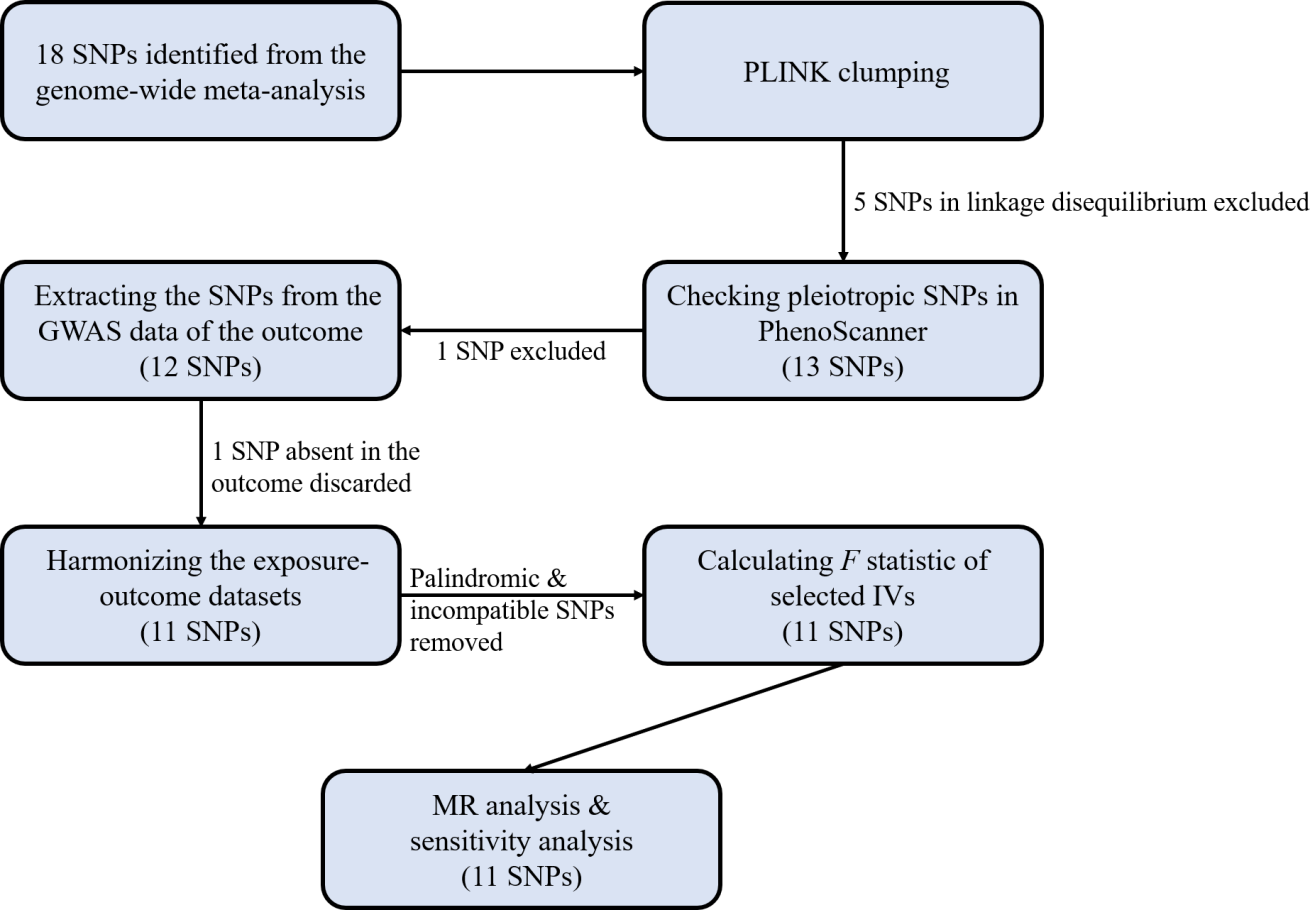
The flowchart of the selection of instrumental variables (IVs).

SNPs robustly associated with circulating Hcy levels at the genome-wide significance threshold (*p* < 5 × 10^−8^) (18 SNPs) were identified from the genome-wide meta-analysis on total Hcy concentrations (*n* = 44,147 participants) in individuals of European ancestry (**Supplementary Table S1**) (49). These SNPs explained approximately 6.0% of the variability observed in Hcy levels. To ensure the independence of the selected SNPs, the PLINK clumping approach was used to exclude SNPs in linkage disequilibrium at a threshold of *r* ^2^ <0.001 within a window size of 10,000 kb in 1000 Genomes data (Europeans) (50). Each of the remaining SNPs was subsequently checked for secondary phenotypes in PhenoScanner (http://www.phenoscanner.medschl.cam.ac.uk) to assess whether these SNPs were in relation to other traits that may be potential confounders at a genome-wide significance level (*p* < 5 × 10^−8^) (51). The SNPs were then extracted from the GWAS data of the outcome (psoriasis). In this step, the SNPs related to psoriasis at genome-wide significance (*p* < 5 × 10^−8^) and those absent in the outcome data (only one SNP) were discarded. After that, the exposure–outcome datasets were harmonized to remove any palindromic SNPs with minor allele frequencies above 0.3 and any incompatible SNPs. Finally, the *F*-statistics of the ultimately selected SNPs were calculated to assess the strengths of the selected IVs using the formula *F* = *R* ^2^ (*n* − 1 − *k*)/(1 − *R* ^2^)*k*. Here, “*n*” denotes the sample size, “*k*” refers to the count of IVs, and “*R* ^2^” represents the proportion of variation explained by the SNPs. IVs with an *F*-statistic greater than 10 were regarded to be strongly associated with the exposure of interest (52). **Figure 2** illustrates the flowchart of IV selection.

### 2.4 MR analyses and sensitivity analyses

The random-effect inverse-variance weighted (IVW) method was employed as the primary analysis to evaluate the cause relationship between circulating Hcy levels and psoriasis, supplemented by the weighted median and MR-Egger regression methods. The weighted median method provides reliable evidence when no more than 50% of instruments are invalid (53). The MR-Egger regression approach allows a consistent causal estimate even if the genetic IVs are all invalid (54). For MR analyses, statistical significance was set at a two-sided *p*-value less than 0.05. To assess any bias of the MR assumptions, sensitivity analyses were performed for the recognized significant MR estimates (IVW *p* < 0.05). Cochrane’s *Q* value was adopted to evaluate the heterogeneity among the estimates of SNPs (55). Cochrane’s *Q*-derived *p* < 0.05 and *I* ^2^ > 25% were considered heterogeneous. Additionally, the MR pleiotropy residual sum and outlier (MR-PRESSO) test was also used to detect the heterogeneity. To assess horizontal pleiotropy, the intercept in MR-Egger regression was utilized (56). Furthermore, leave-one-out analysis was employed to detect whether there existed any SNP having an independent influence on the overall results.

All statistical analyses were executed adopting the “TwoSampleMR” package (version 0.5.7) (https://github.com/MRCIEU/TwoSampleMR) and the “MR-PRESSO” package (version 1.0) in R software (version 4.3.1). No ethical approval was required since the data used in this study were publicly available.

## 3 Results

### 3.1 IVs selection

Originally, 18 SNPs robustly associated with plasma Hcy levels were identified from the genome-wide meta-analysis on circulating Hcy concentrations (49). After clumping, five SNPs in linkage disequilibrium were excluded. Checking each of the remaining 13 SNPs in PhenoScanner, we removed one SNP (rs548987) with potential confounding secondary phenotypes of body mass index and whole body fat mass since there has been an established association between obesity and psoriasis (57, 58). Extracting the residual SNPs from the GWAS data of psoriasis yielded a total of 11 SNPs with 1 SNP (rs234709) discarded because of the absence of this SNP in data of the outcome of psoriasis. All SNPs displayed *F*-statistics exceeding 10, suggesting they were strong instruments. **Table 1** shows the characteristics of the total 11 SNPs included in our analysis.

**Table 1:** Characteristics of SNPs for homocysteine and their association with psoriasis.

					Homocysteine				Psoriasis		
SNP	Chr	EA	OA	EAF	*β*	SE	*p*	*F-*statistic	*β*	SE	*p*
rs12780845	10	A	G	0.65	0.0529	0.009	7.80E−10	56.28	−0.0010	0.017	0.953
rs154657	16	A	G	0.47	0.0963	0.007	1.74E−43	204.90	0.0008	0.015	0.959
rs1801133	1	A	G	0.34	0.1583	0.007	4.34E−104	502.12	−0.0085	0.018	0.632
rs1801222	10	A	G	0.34	0.0453	0.007	8.43E−10	40.69	−0.0434	0.016	0.007
rs2251468	12	A	C	0.65	−0.0512	0.007	1.28E−12	52.72	−0.0035	0.015	0.820
rs2275565	1	T	G	0.21	−0.0542	0.009	1.96E−10	43.07	0.0265	0.018	0.147
rs42648	7	A	G	0.40	−0.0395	0.007	1.97E−08	33.09	−0.0089	0.015	0.563
rs4660306	1	T	C	0.33	0.0435	0.007	2.33E−09	36.97	0.0117	0.016	0.455
rs7130284	11	T	C	0.07	−0.1242	0.013	1.88E−20	88.84	−0.0086	0.022	0.694
rs838133	19	A	G	0.45	0.0422	0.007	7.48E−09	38.95	−0.0082	0.015	0.597
rs9369898	6	A	G	0.62	0.0449	0.007	2.17E−10	41.98	−0.0087	0.015	0.569

SNP, single nucleotide polymorphism; Chr, chromosome; EA, effect allele; OA, other allele; EAF, effect allele frequency; β, per allele effect on exposures; SE, standard error; p, p-value for the genetic association.

### 3.2 MR and sensitivity analyses

**Figure 3:**
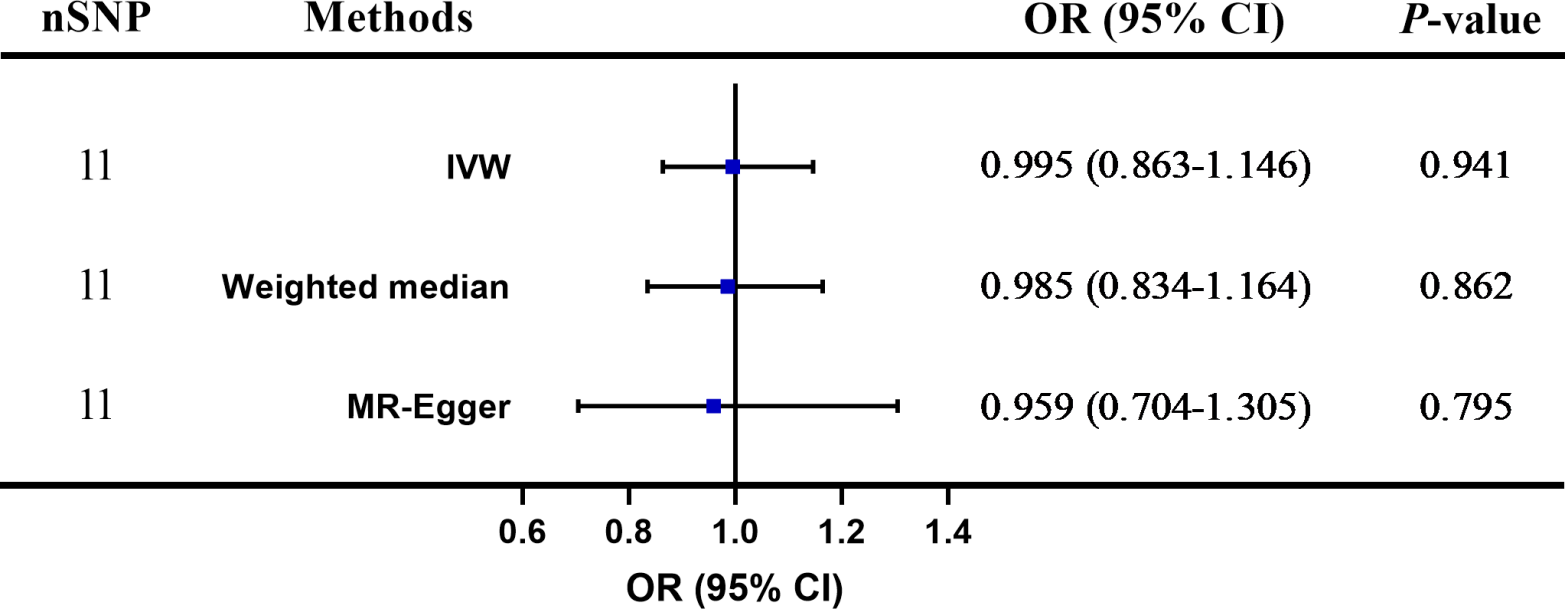
The two-sample Mendelian randomization (TSMR) of plasma homocysteine (Hcy) levels and psoriasis. SNP, single nucleotide polymorphism; IVW, inverse-variance weighted; OR, odds ratio; CI, confidence interval.

**Figure 4:**
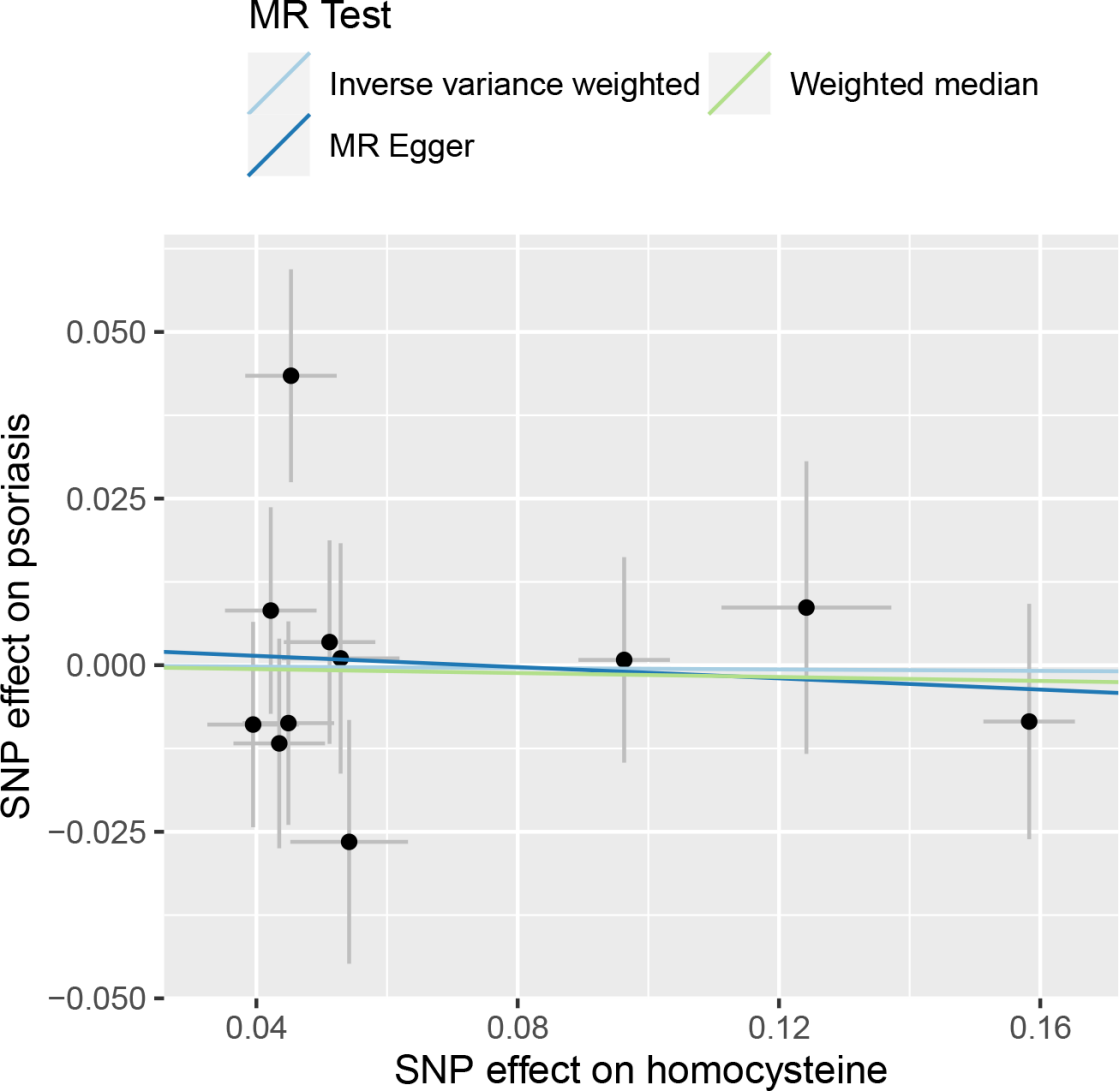
Scatter plot of the causal relationships between homocysteine and psoriasis using different MR methods.

**Figure 3** shows that no significant association existed between Hcy and psoriasis utilizing all 11 SNPs as IVs via MR analysis. As indicated by the random-effect IVW method, the odds ratio (OR) and 95% confidence interval (CI) for each unit rise in circulating Hcy levels within psoriasis were 0.995 (0.863–1.146), *p* = 0.941, which was consistent with the results of the weighted median method [OR = 0.985 (0.834–1.164), *p* = 0.862] and the MR-Egger regression method [OR = 0.959 (0.704–1.305), *p* = 0.795]. A scatter plot depicting the effect sizes of SNPs for Hcy is shown in **Figure 4**.

**Figure 5:**
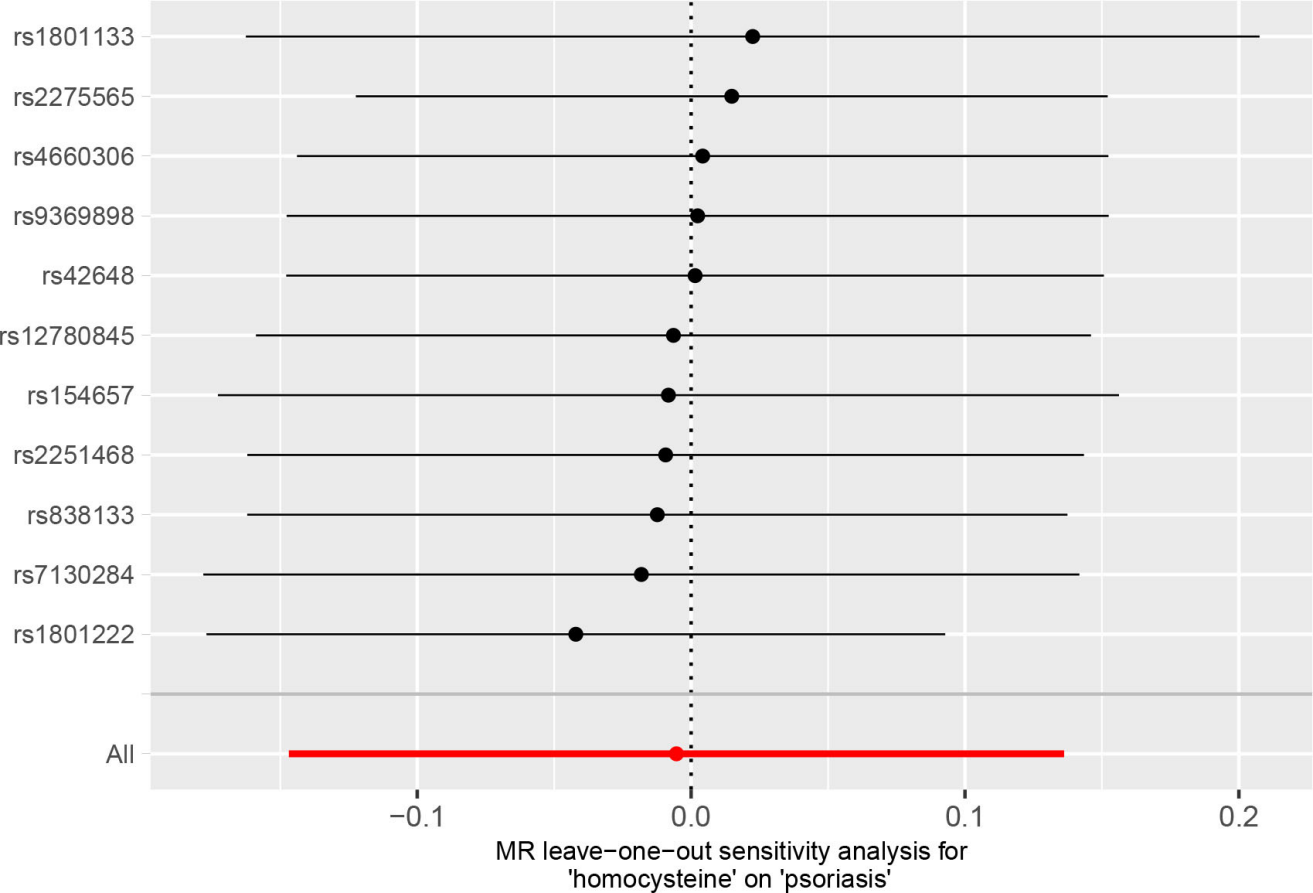
Leave-one-out analysis for the association of plasma homocysteine (Hcy) level with psoriasis.

**Table 2** presents the results of the sensitivity analysis. Cochrane’s *Q* test and MR-PRESSO test showed no evidence of heterogeneity. The *p*-value (*p* > 0.05) for the MR-Egger intercept indicated no directional pleiotropy. Leave-one-out analysis revealed that the causal estimations of Hcy levels were not influenced by any individual SNP, further validating the lack of association between Hcy and psoriasis (**Figure 5**).

**Table 2:** Results of the sensitivity analysis.

Heterogeneity				Pleiotropy	
Cochrane’s *Q*	*I* ^2^	*p*-value for Cochrane’s *Q* test	*p*-value for the MR-PRESSO test	MR-Egger intercept	*p*-value for the MR-Egger intercept
11.432	12.5%	0.325	0.439	0.003	0.795

MR, Mendelian randomization; MR-PRESSO, Mendelian randomization pleiotropy residual sum and outlier.

## 4 Discussion

To our knowledge, this is the first study to investigate the causal effect of plasma Hcy levels on psoriasis from a genetic view through MR analysis methods. Although randomized controlled trials (RCTs) represent the most potent approach for establishing causal hypotheses in epidemiological research, their implementation demands a more stringent research design and incurs higher costs, thereby rendering their execution challenging. MR is a novel epidemiological research method to explore the causal relationship between exposure and outcome. Integrating MR into traditional epidemiology offers a clever solution to address the limitations of conventional research in uncovering causation, including issues related to confounding factors and uncertain causal pathways. This amalgamation opens up novel perspectives and methodologies for investigating the origins of diseases in epidemiological research (59). As the genetic makeup of offspring is inherited in a random manner from their parents, utilizing SNP as a genetic variable tool becomes a highly dependable approach for inferring causal relationships between two factors (31, 32). Consequently, in recent years, some researchers have regarded MR research as the most favorable substitute for RCTs (60). In our research, we investigated the causation between plasma Hcy levels and psoriasis using datasets from large-scale GWASs. The outcomes of our TSMR analysis did not demonstrate a detrimental effect of genetically increased Hcy levels on psoriasis risk. Our results were robust, achieved through the utilization of diverse instrumental variables and analytical methods.

Numerous studies have exhibited a link between elevated plasma Hcy levels and psoriasis. A case–control study found that the average levels of serum total Hcy were elevated in patients with psoriasis and suggested that increased plasma Hcy concentrations might significantly contribute to the emergence of atherothrombotic complications in individuals with psoriasis (16). Brazzelli et al. additionally documented a notably greater occurrence of hyperhomocysteinemia in individuals with psoriasis compared with the healthy controls (19). Similarly, the same findings were discovered in a study conducted by Malerba et al., where a connection between Hcy levels and disease severity was also reported. They suggested that individuals suffering from psoriasis could be potentially inclined to experience hyperhomocysteinemia, a condition that might make them more susceptible to increased cardiovascular risks (17). Additionally, two studies also indicated the association of plasma Hcy concentrations with the severity of psoriasis (22, 23). However, using an MR analysis approach, our findings did not demonstrate a direct causal effect of plasma Hcy levels on psoriasis. Consistent with our results, it was reported by Uslu et al. that no statistically significant differences existed between psoriatic patients and the control group in terms of plasma Hcy levels (*p* > 0.05) (27). Similar results were also found in a case–control study conducted by Liew et al. (26). Additionally, including individuals with moderate to severe plaque psoriasis (in the patient group) and those with non-psoriatic dermatological conditions (as controls), Akcali et al. documented the absence of any disparity in homocysteine levels between these two groups (29). Furthermore, two more studies by Cakmak et al. and Erturan et al., involving patients with a shared ethnic background, consistently reported the absence of a significant elevation in plasma Hcy levels among individuals with psoriasis (28, 30). These research works confirmed our research results.

With regard to the observational studies where high plasma homocysteine levels were noticed in patients with psoriasis, caution should be observed when interpreting the research findings especially the relationship between Hcy and psoriasis. Hcy, an amino acid derived from methionine, is catabolized to cysteine with the contribution of vitamin B_12_ and folate (22). Previous studies suggested that high levels of homocysteine in patients with psoriasis were attributed to deficiencies in folate and vitamin B_12_ rather than a direct effect of psoriasis (17, 19). Decreased folate absorption may result from vitamin consumption by the skin or intestinal inflammation (17). Tobin et al. suggested that keratinocytes of patients with psoriasis regenerated faster, during which folate may be utilized for methylating DNA and undergoing mitotic activity, thus increasing homocysteine levels (20, 24). Additionally, previous research found that homocysteine might facilitate the progression of psoriasis by driving immune inflammatory processes through some pathways, such as activating Th1 and Th17 cells and inducing the activation of nuclear factor κB (25). This kind of possible promotion of psoriasis progression may not mean that Hcy could causally give rise to the occurrence of psoriasis. Although some studies showed the correlation between Hcy and psoriasis, the causal relationship between them has not been proven. Whether Hcy exerts a causative role in the initiation of psoriasis remains unclear.

Furthermore, considering that Hcy has been documented as an independent risk factor for cardiovascular diseases (14, 15), caution should be observed when performing observational studies to explore the association between plasma Hcy levels and psoriasis. It is important to note that psoriatic patients with cardiovascular diseases or risk factors may not be included in the research. The issue of the association and causation between Hcy and psoriasis requires further study.

There were several limitations in our study. First, although we have employed the PhenoScanner to check for secondary phenotypes of potential confounders and excluded them, there still existed a possibility that we did not entirely eliminate the impact of potential pleiotropy, through which the IVs influence the outcome of psoriasis. Second, the predominant population included in our analysis is of European ancestry. Thus, caution should be observed when interpreting the results among different ancestries. Third, we investigated the causal relationship between Hcy and psoriasis from a genetic view. To comprehensively understand the pathogenesis of psoriasis, environmental factors should also be taken into account.

## 5 Conclusion

To conclude, our study findings did not demonstrate a causal effect of genetically determined circulating Hcy levels on psoriasis utilizing a genetic approach. Confirmation of the findings from this study necessitates a further MR analysis, utilizing a more extensive dataset from GWASs and incorporating a greater number of genetic instruments. Future research is needed to further explore the mechanism of the association between Hcy and psoriasis.

## Data availability statement

The original contributions presented in the study are included in the article/**Supplementary Material**. Further inquiries can be directed to the corresponding author.

## Author contributions

CC: Conceptualization, Formal Analysis, Investigation, Methodology, Software, Visualization, Writing – original draft. SL: Data curation, Writing – original draft, Investigation. JL: Formal Analysis, Visualization, Writing – original draft. ZZ: Formal Analysis, Visualization, Writing – original draft. YZ: Software, Validation, Writing – review & editing. ZL: Software, Validation, Writing – review & editing. YL: Conceptualization, Funding acquisition, Supervision, Writing – review & editing.
