# Supplementary material for: No causal effect of genetically determined circulating homocysteine levels on psoriasis in the European population: evidence from a Mendelian randomization study
Source: Front Immunol. 2023 Nov 8;14:1288632. doi: 10.3389/fimmu.2023.1288632 (PMC10663369; doi:10.3389/fimmu.2023.1288632)
Supplement: Supplementary file 1 [file Table_1.docx]

Supplementary Material

**Table S1. Characteristics of SNPs for homocysteine.**

| SNP | Chromosome | Nearest gene | EA | OA | EAF | *β* | SE | *p* |
| --- | --- | --- | --- | --- | --- | --- | --- | --- |
| rs12134663 | 1 | *MTHFR* | A | C | 0.8 | -0.101 | 0.011 | 2.54E-21 |
| rs12780845 | 10 | *CUBN* | A | G | 0.65 | 0.0529 | 0.009 | 7.80E-10 |
| rs12921383 | 16 | *DPEP1/FANCA* | T | C | 0.87 | -0.09 | 0.014 | 8.22E-11 |
| rs154657 | 16 | *DPEP1* | A | G | 0.47 | 0.0963 | 0.007 | 1.74E-43 |
| rs1801133 | 1 | *MTHFR* | A | G | 0.34 | 0.1583 | 0.007 | 4.34E-104 |
| rs1801222 | 10 | *CUBN* | A | G | 0.34 | 0.0453 | 0.007 | 8.43E-10 |
| rs2251468 | 12 | *HNF1A-AS1* | A | C | 0.65 | -0.0512 | 0.007 | 1.28E-12 |
| rs2275565 | 1 | *MTR* | T | G | 0.21 | -0.0542 | 0.009 | 1.96E-10 |
| rs234709 | 21 | *CBS* | T | C | 0.45 | -0.0718 | 0.007 | 3.90E-24 |
| rs2851391 | 21 | *CBS* | T | C | 0.47 | 0.056 | 0.008 | 1.70E-12 |
| rs42648 | 7 | *GTPB10* | A | G | 0.4 | -0.0395 | 0.007 | 1.97E-08 |
| rs4660306 | 1 | *MMACHC* | T | C | 0.33 | 0.0435 | 0.007 | 2.33E-09 |
| rs548987 | 6 | *SLC17A3* | C | G | 0.13 | 0.0597 | 0.01 | 1.12E-08 |
| rs7130284 | 11 | *NOX4* | T | C | 0.07 | -0.1242 | 0.013 | 1.88E-20 |
| rs7422339 | 2 | *CPS1* | A | C | 0.33 | 0.0864 | 0.008 | 4.58E-27 |
| rs838133 | 19 | *FUT2* | A | G | 0.45 | 0.0422 | 0.007 | 7.48E-09 |
| rs9369898 | 6 | *MUT* | A | G | 0.62 | 0.0449 | 0.007 | 2.17E-10 |
| rs957140 | 11 | *NOX4* | A | G | 0.45 | -0.045 | 0.008 | 2.43E-08 |

SNP, single nucleotide polymorphism; EA, effect allele; OA, other allele; EAF, effect allele frequency; *β*, per allele effect on exposures; SE, standard error; *p*, *p* value for the genetic association.
